# RODFormer: High-Precision Design for Rotating Object Detection with Transformers

**DOI:** 10.3390/s22072633

**Published:** 2022-03-29

**Authors:** Yaonan Dai, Jiuyang Yu, Dean Zhang, Tianhao Hu, Xiaotao Zheng

**Affiliations:** Hubei Provincial Engineering Technology Research Center of Green Chemical Equipment, School of Mechanical and Electrical Engineering, Wuhan Institute of Technology, Wuhan 430205, China; 11901010003@stu.wit.edu.cn (Y.D.); 22002010043@stu.wit.edu.cn (D.Z.); 22002010036@stu.wit.edu.cn (T.H.); xiaotaozheng@wit.edu.cn (X.Z.)

**Keywords:** rotating object detection, structured transformers, spatial-FFN, RODFormer

## Abstract

Aiming at the problem of Transformers lack of local spatial receptive field and discontinuous boundary loss in rotating object detection, in this paper, we propose a Transformer-based high-precision rotating object detection model (RODFormer). Firstly, RODFormer uses a structured transformer architecture to collect feature information of different resolutions to improve the collection range of feature information. Secondly, a new feed-forward network (spatial-FFN) is constructed. Spatial-FFN fuses the local spatial features of 3 × 3 depthwise separable convolutions with the global channel features of multilayer perceptron (MLP) to solve the deficiencies of FFN in local spatial modeling. Finally, based on the space-FFN architecture, a detection head is built using the CIOU-smooth L1 loss function and only returns to the horizontal frame when the rotating frame is close to the horizontal, so as to alleviate the loss discontinuity of the rotating frame. Ablation experiments of RODFormer on the DOTA dataset show that the Transformer-structured module, the spatial-FFN module and the CIOU-smooth L1 loss function module are all effective in improving the detection accuracy of RODFormer. Compared with 12 rotating object detection models on the DOTA dataset, RODFormer has the highest average detection accuracy (up to 75.60%), that is, RODFormer is more competitive in rotating object detection accuracy.

## 1. Introduction

Object detection is the core task in the field of computer vision and the basis for object tracking and behavior recognition [1]. Objects in any direction are widely distributed in application scenarios, such as scene text detection and pipeline object detection. Because the objects are always small, inclined and dense, the detection of directional objects becomes very difficult [2]. Therefore, researchers have proposed many rotating object detection algorithms, including RRPN [3], R^2^CNN [4], RoI-Transformer [5] and SCRDet [6]. Although these methods have achieved good performance, they are sensitive to the super parameters of Anchor and are prone to the problem of loss discontinuity, thus leading to the decline of object detection accuracy.

Considering the complexity of detecting objects and a large number of small and stray rotating objects, the DETR model constructed by Carion et al. [7] was the first to apply Transformer to the field of object detection. Transformer is a fully attention-based encoder–decoder model built by Vaswani et al. [8]. DETR directly detects all objects by introducing object queries, achieving true end-to-end detection with less feature information. However, during the initialization process of the transformers, each query uses the same weights for all positions, which makes the training volume of the model very large. In order to solve this problem, Zhu et al. [9] sparsely sampled dense keys in the attention operation so that each query only needs to aggregate the sparse keys and reduce the computational cost of the model. Dai et al. [10] constructed the UP-DETR model. UP-DETR pretrained the object query in DETR with a new pretext task-multi-query localization and improved the convergence speed of transformers in DETR with unsupervised pretraining. Although DETR and its improved algorithm effectively improve the training effect of the model, the transformer contains a feed-forward network (FFN) [11], and FFN is composed of MLP. Compared with convolutional layers, FFNs are more efficient and can model better long-term dependencies and position patterns. However, the fully connected layer is based on the global receptive field of the channel. For the detection of small objects, if the fully connected layer structure is still used, the object to be detected is submerged in the background average feature, which causes MLP to lack local space modeling ability [12].

To improve Transformer’s ability to extract local information, researchers improved DETR from three aspects: sparse attention [9], spatial prior [13] and structural redesign [14]. For example, Ding et al. [15] proposed the RepMLP model for image recognition. RepMLP utilizes the local spatial-modeling capability of CNN to improve the local information-collection capability of RepMLP. Beal et al. [16] combined ViT and RPN to construct ViT-FRCNN and verified for the first time that transformers as the backbone can be directly applied to images and maintain good classification effect. Wang et al. [17] proposed the pyramid vision transformers (PVT) model. PVT uses global down-sampling to design the global subsampled attention (GSA). Liu et al. [18] constructed the Swin Transformer model, which constructs the structural backbone as a local-to-global combination, thereby avoiding the quadratic computation of the algorithm and improving the algorithm convergence speed. Although the above methods improve the Transformer’s ability to extract local information to a certain extent, the addition of CNN in RepMLP greatly increases the computational load of the Transformer, and the lack of connections between different windows in Swin Transformer leads to the limitation of the receptive field. At the same time, these methods are based on anchor-base target detection, and anchor base ignores the matching mechanism of extreme samples, which is not in line with the design idea of DNN.

In order to solve the problems existing in anchor, researchers proposed the anchor-free method [18]. Compared with anchor base, the biggest advantage of anchor-free is the detection speed. Anchor-free does not require preset anchors but only needs to regress the object center point, width and height of the feature map, which improves the information collection of the model and alleviates the discontinuity problem of the boundary loss of the rotating frame. For example, SCRDet introduces an IoU constant factor in the SmoothL1 loss [19] to alleviate the boundary problem of the rotated box. Zhao et al. [20] proposed a different polar coordinate method (PolarDet), which uses center-point positioning, orients with four polar angles and measures with a polar ratio system to further improve the accuracy of object detection. Han et al. [21] proposed a single-shot alignment network (S^2^A-Net) that employs an active rotation filter to encode orientation information to alleviate the inconsistency between classification scores and localization accuracy.

In view of Transformer’s lack of local space modeling capabilities, we provide an effective hybrid architecture to further improve object detection accuracy, and the architecture is spatial-FFN. Spatial-FFN combines the local space characteristics of 3 × 3 depth separable convolution with the global channel characteristics of MLP. Using structured transformer-level module (STS), Spatial-FFN module (STS) and CIOU-smooth L1 loss function (C-SL1) as the ablation experimental modules of RODFormer, the results show that the three modules in RODFormer (STS, STS and C-SL1) are effective for improving the object detection accuracy of the model. On the DOTA [22] dataset, comparing RODFormer with 12 rotating object detection models, RODFormer has the highest *mAP* value, that is, RODFormer has the best object detection effect.

## 2. Methods

**Figure 1:**
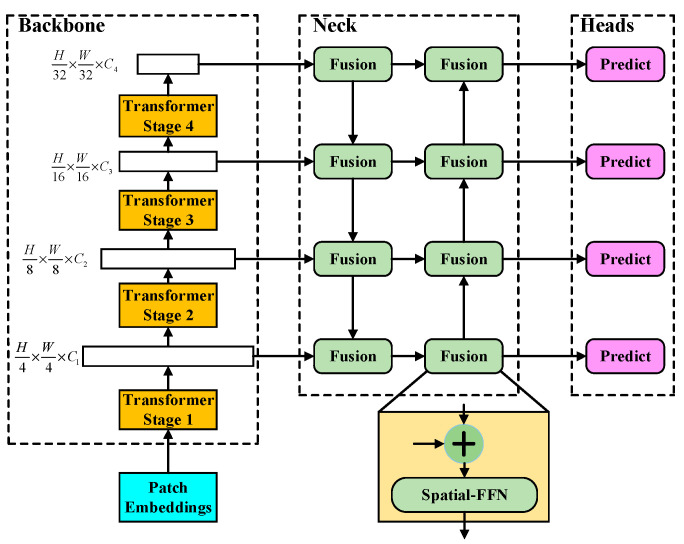
Structure of RODFormer.

The RODFormer framework is shown in Figure 1. RODFormer is mainly composed of backbone, neck and head. Firstly, RODFormer’s backbone structure uses structured transformers to extract the features of images. Secondly, the obtained multi-level features are input into the neck structure and enhanced by the constructed spatial-FFN structure to solve the lack of spatial local modeling ability of FFN. Finally, the enhanced multi-level features are transferred to the head structure, and the first-order, anchor-free, eight-parameter regression method is used to predict and detect the image, which reduces the complexity of the second-order structure and alleviates the loss discontinuity of the rotating frame.

### 2.1. Backbone

(1) Structured design

**Figure 2:**
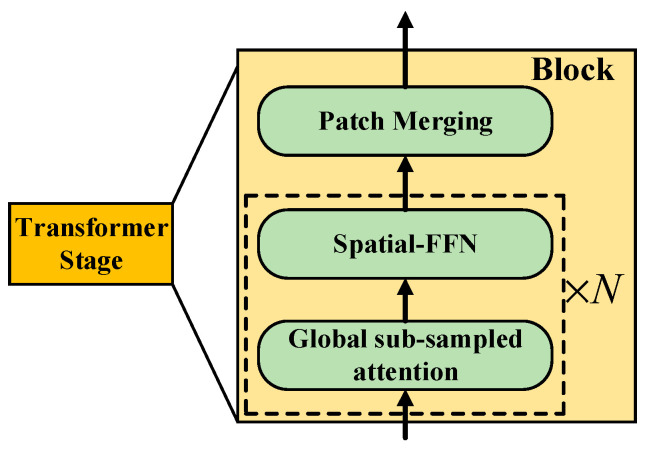
Structure of block.

Unlike ViT, which uses patches of 16 × 16 size all the time, using smaller patches is beneficial to the prediction task of dense small objects. For images of *H* × *W* × 3 size, RODFormer constructs four stages with different resolutions to realize the structuring of transformers and divides the input images by patch partition to obtain patches of (4 × 4) size from stage 1 to stage 4, with the following feature resolutions: $\frac{H}{2_{i+1}}\times \frac{W}{2_{i+1}}\times C_{i}$,  i∈{1, 2, 3, 4}, *C_i+_*_1_ is greater than *C_i_*. Each stage consists of blocks with the same structure but different numbers (the specific parameters are shown in Table 1 in Section 2.1). The structure of each block is shown in Figure 2.

(2) Global subsampled attention

Traditional transformers object detection adopts the self-attention model [23]. The long-distance information-capture ability of self-attention is comparable to that of RNN and far exceeds that of CNN. Among them, self-attention includes scaled dot-product attention (Equation (1)) and multi-head attention (Equation (2)).

(1) $$\mathrm{Attention}\;\left(Q,K,V\right)=\mathrm{softmax}\left(\frac{K^{T}Q}{\sqrt{d_{k}}}\right)V$$

MultiHead (*Q*, *K*, *V*) = Concat (head_1_, …, head_h_)*W^O^* (2)

where, *Q*, *K*, *V* are query vector sequence, key vector sequence and value vector sequence, respectively. head*_i_* = Attention (*QW_i_^Q^*, *KW_i_^K^*, *VW_i_^V^*). $\sqrt{d_{k}}$ is to scale the inner product to avoid softmax results from either 0 or 1.

To further add global attention and reduce the complexity of self-attention, the easiest way is to add a global attention layer after each local attention block so that information can be exchanged across windows, with time complexity ($O({\left(k_{1}\times k_{2}\times m\times n\right)}^{2}\times d)$) also increased further. Therefore, RODFormer adopts the global subsampled attention (GSSA) method [24,25] to reduce the time complexity ($O(\frac{H^{2}W^{2}d}{k_{1}k_{2}}+k_{1}k_{2}HWd)$) of the whole process (the process of local and global attention) through the subsampling function. Among them, *m* and *n*, represented as feature maps, are divided into (*m* × *n*) subwindows, *k*_1_ = *H*/*m*, *k*_2_ = *W*/*n*.

Based on Ref. [17], the length of the sequence was reduced using the reduction ratio (*R*) shown below.

*K*′ = Reshape (*N*/*R*^2^, *C*·*R*) (*K*)
*K* = Linear (*C*·*R*^2^, *C*) (*K*′) (3)

where, *K*’s sequence is to reduce, and the dimension of the new *K* is *N*/*R* × *C*, since the complexity of the attention mechanism is reduced from *O* (*N*^2^) to *O* (*N*^2^/*R*^2^). From stage 1 to stage 4, set *R* to [1,2,4,8].

(3) Spatial-FFN

Because the resolution of PE is fixed, when the resolution is layered, the position code needs to be interpolated; however, this will lead to a decrease in accuracy. Each layer in the encoder and decoder in the traditional Transformers contains a channel-based global modeling FFN (Equation (4)). The FFN consists of two linear transformations and is activated by the ReLU function. CNN obtains the global feature information of the image through local perception (Equation (5)).

FFN(*x*) = max (0, *xW*_1_ + *b*_1_)*W*_2_ + *b*_2_ (4)

(5) $$\mathrm{CNN}(x)=f(\sum_{i}w_{i}x_{i}+b)$$

where, *W*_1_ and *W*_2_ are weight matrices, and *b*_1_ and *b*_2_ are bias vectors. *f* represents the nonlinear function, and *w* and *b* represent the weight and bias of the fully connected layer, respectively. *x* represents the feature of the input.

**Figure 3:**
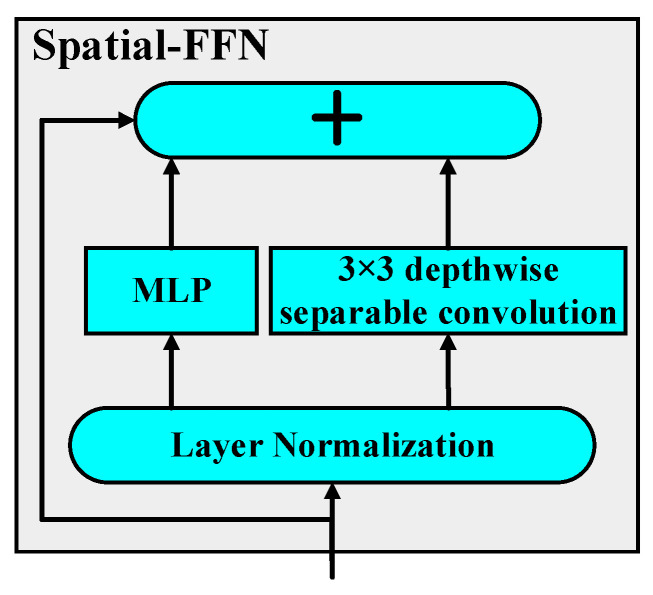
Structure of spatial-FFN.

However, the attention in Transformers is position-invariant and requires position embedding to determine feature information. At the same time, FFN lacks spatial local modeling capabilities, and CNN adds complexity to the network structure. Therefore, RODFormer introduces spatial-FFN, which integrates the global capability of FFN and the local capability of 3 × 3 depthwise separable convolution [26] by means of upsampling through layer normalization [27]. Because point-wise convolution and position-wise FFN are equivalent, introducing spatial-FFN to each Transformers block enables modeling of local spatial relationships through the network. The built-in properties of spatial-FFN allow for the removal of location information in the network without affecting performance, which both strengthens the local modeling effect of the network and avoids the structural complexity of CNNs. The structure of spatial-FFN is shown in Figure 3.

In Figure 3, + stands for upsampling. Layer normalization (LN) normalizes different channels of the same sample. Because LN is an algorithm independent of batch size, the number of samples will not affect the amount of data involved in LN calculation. The expression of LN is as follows:

(6) $$h=f(\frac{g}{\sqrt{\sigma^{2}+\varepsilon }}\cdot (a-\mu )+b)$$

where *σ* and *μ* are the normalized statistics of LN, *a* is the normalized value of LN, *g* is the gain and *ε* prevents the occurrence of division by 0.

(4) Patch merging

For each patch of the image, ViT uses the patch-merging method to unify the N × N × 3 patches into a 1 × 1 × C vector to obtain a hierarchical feature map. However, ViT is used to combine non-overlapping feature blocks and cannot maintain the local continuity of patches. Therefore, according to reference [28], the overlapping patch-merging method is used to convert the feature dimensions of different patches into C1, C2, C3 and C4, respectively, and then send them into the Transformers block.

### 2.2. Neck and Heads

(1) Neck

**Figure 4:**
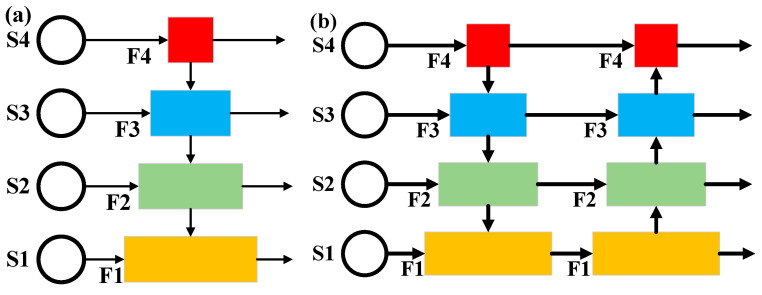
Structure of Neck. (**a**) PANet structure; (**b**) Bidirectional fusion structure.

Neck is used to collect and strengthen different feature maps, and a neck consists of multiple bottom-up paths and top-down paths. The PANet structure adopts bottom-up path augmentation to recover the corrupted information between each proposal region and all feature levels through adaptive feature pooling, and its structure is shown in Figure 4a. RODFormer uses the bidirectional fusion method shown in Figure 4b to enhance the feature information of the image. All features adopt the spatial-FFN structure. RODFormer changes the global channel of FFN through spatial-FFN, avoiding the structural complexity of CNN and the limitations of FFN.

(2) Head

**Figure 5:**
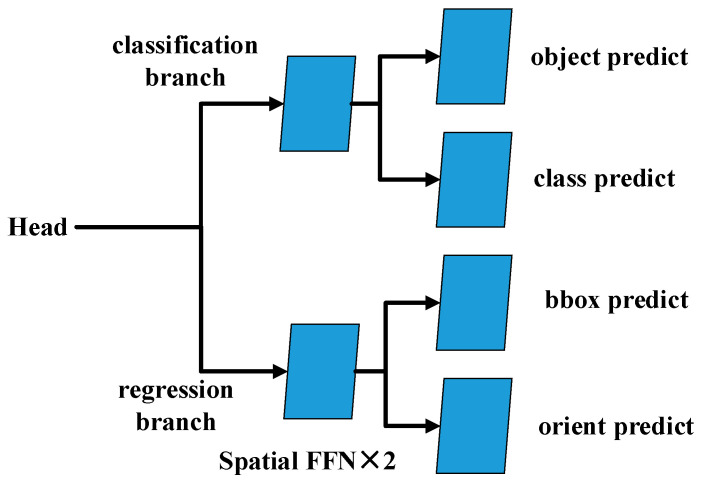
Structure of the head.

Using structured transformers, 4 parallel output predictions are obtained for the 4 layers of transformer stages. Head includes a classification branch and a regression branch, which both use spatial-FFN to predict image features. The structure of the head is shown in Figure 5. The classification branch is mainly used for the classification of objects and object categories, and the regression branch is mainly used for the regression processing of the rotation frame. To reduce the complexity of the structure, RODFormer adopts a first-order, anchor-free mode, which reduces the prediction of each position from 3 to 1. RODFormer directly predicts the two offsets of the grid center point, as well as the height and width of the predicted box.

**Figure 6:**
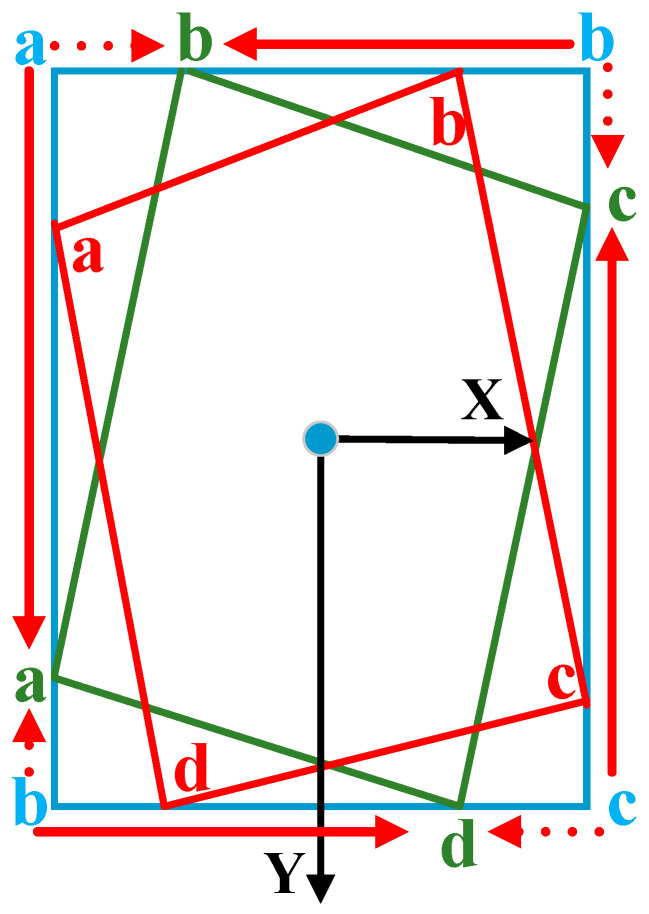
Structure of eight-parameter regression.

RODFormer adopts the eight-parameter regression method [29]. In addition to the basic four-regression point classification, this definition method also includes four regression points of the horizontal box. The upper-left corner of the definition is the starting point, and the remaining points are arranged in counterclockwise order, as shown in Figure 6. To solve the loss discontinuity of the rotation box, when the coincidence ratio of the rotation box area and the original horizontal box area is close to 1, only the horizontal box is regressed.

To solve the problem of the discontinuity of the loss of the rotating box, when the coincidence ratio of the area of the rotating box and the area of the original horizontal box is close to 1, the frame only reverts to the horizontal box. Since this paper predicts remote sensing rotation images under the framework of Transformers, the prediction is not an ordered set like the traditional object detection result but an unordered set. To limit the gradient value, the horizontal box of RODFormer adopts the CIOU loss function (the coefficient is 2) (Equation (7)) [30], and the rotation box adopts Smooth L1 as the loss function (Equation (8)) [19].

(7) $$L_{\mathrm{CIOU}}=1-IOU(A,B)+\rho^{2}(A_{ctr},B_{ctr})/c^{2}+\alpha v\;v=\frac{4}{\pi^{2}}{\left(\arctan\frac{w^{gt}}{h^{gt}}-\arctan\frac{w}{h}\right)}^{2},\;\alpha =\frac{v}{(1-IoU)+v}$$

(8) $$\mathrm{Smooth}L1(x)=\{\begin{array}{c} 0.5x{{}^{\prime }}^{2}if\left|x{}^{\prime }\right|<1\\ \left|x{}^{\prime }\right|-0.5\mathrm{otherwise} \end{array}$$

where, *A* and *B* are the predicted frame and the real frame, respectively; *A_ctr_* is the coordinates of the center point of the prediction frame; *B_ctr_* is the coordinates of the center point of the real frame; *ρ* is the Euclidean distance calculation; *c* is the diagonal length of the minimum bounding box of *A* and *B*; *w^gt^* and *h^gt^* represent the width and height of the real box, respectively; *w* and *h* represent the width and height of the predicted box, respectively; and *x*′ is the elementwise difference between *A* and *B*.

## 3. Experiments

In this section, we demonstrate the effectiveness of RODFormer on the commonly used, publicly available aerial dataset (DOTA). After introducing the experimental settings and evaluation metrics, RODFormer is compared with state-of-the-art models.

### 3.1. Datasets

The current main object detection datasets are shown in Table 1. It can be seen from Table 1 that the DOTA dataset collects the smallest images (10–50 pixels), and this paper mainly detects small objects, so the DOTA dataset is used as the training sample.

Since the sizes of various objects in the DOTA dataset are very different, this brings great trouble to the training of RODFormer. For example, a boat can be as small as 40 pixels, and a bridge can be as large as 1200 pixels. At the same time, various objects in the DOTA dataset also have the characteristics of high spatial resolution and large aspect ratio. Therefore, in order to effectively detect small target objects, before training RODFormer, the DOTA dataset needs to be cropped to obtain images of the same size.

The DOTA dataset consists of 2806 aerial images with 188,282 instance objects annotated by horizontal boxes and rotated boxes. The DOTA dataset has 15 common detection object categories, including plane (PL), baseball diamond (BD), bridge (BR), ground track field (GTF), small vehicle (SV), large vehicle (LV), ship (SH), tennis court (TC), basketball court (BC), storage tank (ST), soccer ballfield (SBF), roundabout (RA), harbor (HA), swimming pool (SP) and helicopter (HC). The training set, validation set and test set in the DOTA dataset comprise 1411, 458 and 937 entries, respectively. The size of the images is between 800 × 800 pixel and 4000 × 4000 pixels. Due to the large variation range of objects in the DOTA dataset (10–50 pixels), testing against the DOTA dataset has more practical application value.

### 3.2. Experimental Environment and Evaluation Index

The experimental environment is shown in Table 2. According to Ref. [22], RODFormer uses average precision (*AP*) and mean average precision (*mAP*) to evaluate the detection accuracy of the model. The total batch size of RODFormer is set to 16, corresponding to 16 images for subtraining. The total number of training epochs, the initial learning rate and the weight decay rate are set to 300, 0.0001 and 0.0001, respectively. The loU threshold for the entire *AP* score is set to 0.1.

### 3.3. Experimental Results and Analysis

Since the images in the DOTA dataset are larger before the training process, the images are cropped to smaller images of (800 × 800) pixels for training. The cropped images are generated with new label information to facilitate future model training. About 28,000 small images are obtained after cropping.

(1) Ablation experiments

Table 3 shows the ablation test results of RODFormer, in which the bold font is the maximum value of each column, and the unit of the values in the table is %. As shown in Table 3, when the basic backbone is ResNet50 and ResNet125, the *mAP* is 63.89% and 66.85%, respectively. When using ViT-B4, the *mAP* is only 69.20%. After adding the STS module, *mAP* increased by 1.18%. After adding the SFM module, *mAP* increases by 3.06% compared to adding the STS module only. Finally, after adding the C-SL1 module, compared with adding the STS module and the SFM module, the *mAP* increases by 2.16%, reaching 75.60%. The results in Table 3 show that after accumulatively adding each module, the *mAP* also gradually increases, that is, the STS module, the SFM module and the C-SL1 module all help to improve the detection accuracy of rotating targets, thus proving the effectiveness of each module.

(2) Comparative Experiment

RODFormer is compared with 12 rotating object detection methods (RRPN [3], R^2^CNN [4], RoI-Transformers [5], CADNet [31], DRN [32], ICN [33], RADet [34], SCRDet [6], MFIAR-Net [22], IRetinaNet [35], PolarDet [20] and S^2^A-Net [21]) to verify its detection accuracy. Table 4 summarizes the detection results of different models for 15 categories in the DOTA dataset.

It can be seen from Table 4 that RODFormer has the best *mAP*. For single-class detection accuracy, RODFormer’s *AP* values are within 0.5% of the best results on PL, TC, and BC. At the same time, the results demonstrate that RODFormer has the highest detection accuracy when detecting small and dense rectangular objects, such as BR, SV, LV and SH, mainly because RODFormer uses depthwise separable convolution and Transformer to effectively combine local and global visual representation information. The clever combination of these two approaches can efficiently encode local and global information. Dense objects have richer local features, which lead to higher accuracy. For the detection of sparse targets, due to the lack of local spatial features, the accuracy of RODFormer is slightly lower than that of other detection networks. Due to the removal of positional embeddings and reduced computational cost, our method outperforms other methods in overall accuracy.

**Figure 7:**
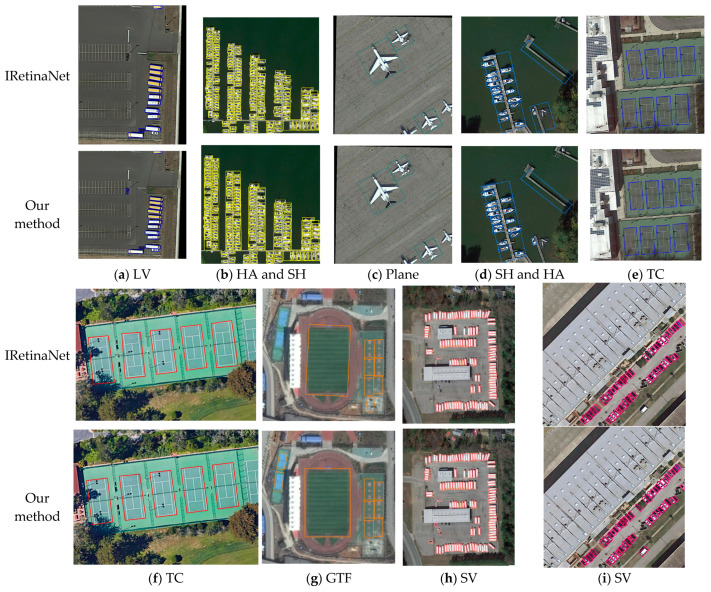
Comparison of RODFormer and IRetinaNet.

**Figure 8:**
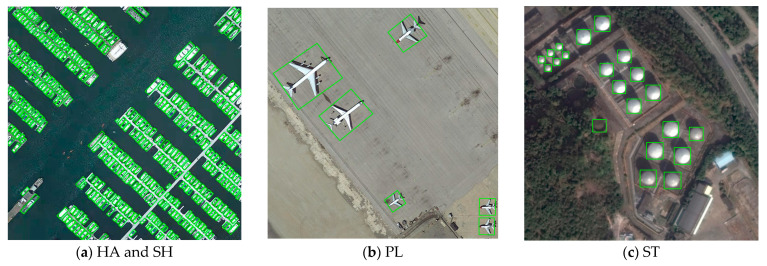

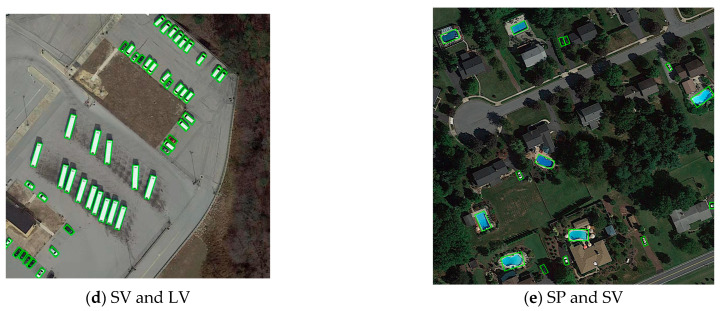
Visualization results of some detected objects.

To visually display the target-detection effect of RODFormer, some image-detection effects of RODFormer and IRetinaNet in the DOTA dataset are visualized, and the results are shown in Figure 7. Figure 8 shows the visualization results of RODFormer in some categories of images in the DOTA dataset.

As can be seen from Figure 7, IRetinaNet is prone to misjudgment on small and dense targets, such as ships, large and small vehicles, etc. On sparse objects, RODFormer has similar accuracy to IRetinaNet, but RODFormer uses an eight-parameter regression method to make the anchor box fit better, such as for ground track fields and tennis courts. Compared with IRetinaNet, RODFormer is superior in comprehensive accuracy, which proves the detection effect of RODFormer on local spatial features and provides ideas for the development of subsequent Transformers.

As shown in Figure 8, this paper presents a small image containing a typical scene, and RODFormer can accurately detect the position of the object. The results show that RODFormer can handle challenging situations, namely rotating objects in high-density or cluttered scenes.

For example, in Figure 8a, the main objects in the image are boats. Since the rotation angles of these objects are almost equal, the use of distance loss suffers from a boundary problem, i.e., it is difficult for the model to distinguish which side is longer. To reduce the loss, RODFormer uses an eight-parameter regression method to synchronously regress anchor box corners. Therefore, we can see that the method can effectively solve the boundary problem and obtain accurate results.

Our method is an improvement on Transformer. To verify the combined effect of these techniques, we conduct ablation experiments and compare the accuracy with that of some other methods. Based on the above experimental results, RODFormer is better than other methods in object detection.

## 4. Conclusions

In this paper, a RODFormer model is proposed for the problem of rotating object detection. RODFormer is composed of Backbone, Neck and Head. Among them, the structure Transformers is used as the backbone to solve the complexity of CNN, and at the same time, the spatial-FFN is designed to solve the problem of the lack of space and local receptive field of FFN. A lightweight detection head is built through the spatial-FFN architecture to alleviate the loss discontinuity problem of the rotating frame. The results of ablation experiments show that the three proposed modules (the structured transformers stage module, the spatial-FFN module and the CIOU-smooth L1 loss function module) all help to improve the detection accuracy of objects. On the DOTA dataset, RODFormer is compared with 12 advanced rotating object detection models. The results show that RODFormer’s *mAP* is the best, and for dense target detection, the accuracy of RODFormer detection is higher because of its richer local features. On the contrary, for sparse object detection, the accuracy of RODFormer detection is slightly behind that of other detection networks due to its insufficient local spatial features. The visual comparison results further demonstrate the good detection effect of RODFormer on the DOTA dataset.

## Figures and Tables

**Table 1:** Comparison of the target size of the dataset.

Dataset	10–50 Pixels	50–300 Pixels	>300 Pixels
DOTA	0.57	0.41	0.02
NWPU VHR-10	0.15	0.83	0.02
MSCOCO	0.43	0.49	0.08
PASCAL VOC	0.14	0.61	0.25

**Table 2:** Experimental environment and parameter settings.

Configure	Setting	Parameter	Setting
Experiment system	Ubuntu 20.04	Backbone	ViT-B4
Learning framework	1.10	Total batch size	16
GPU	Nvidia RTX 3090 Ti	Epoch	300
Initial weight	Xavier init	Initial learning rate	10^−4^
Programming language	Python 3.9	Weight decay rate	0.0001
		Number stage	1, 2, 5, 8
		IoU	0.1

**Table 3:** Results of ablation experiments with RODFormer.

Backbone	STS	SFM	C-SL1	PL	BD	BR	GTF	SV	LV	SH	TC	BC	ST	SBF	RA	HA	SP	HC	*mAP*
			*AP*																
ResNet50	×	×	×	88.03	74.49	38.02	66.34	60.24	46.56	68.20	86.39	77.12	78.28	52.50	61.15	50.82	60.21	49.99	63.89
ResNet152	×	×	×	88.92	77.82	41.50	61.86	67.32	53.97	72.19	89.88	78.65	74.92	53.25	58.41	52.47	68.85	62.78	66.85
ViT-B4	×	×	×	88.21	76.63	45.81	70.25	66.21	71.59	80.69	89.64	80.69	80.25	57.06	58.61	62.93	60.96	48.51	69.20
ViT-B4	√	×	×	89.51	77.58	47.51	69.03	68.94	77.69	82.01	86.50	82.36	82.12	59.08	56.02	58.90	62.38	53.06	70.38
ViT-B4	√	√	×	**89.80**	79.59	48.93	71.43	72.54	80.51	87.95	**90.75**	86.09	**83.69**	60.01	60.39	62.94	68.02	58.98	73.44
ViT-B4	√	√	√	89.76	**79.64**	**56.61**	**71.57**	**78.60**	**85.29**	**89.93**	90.53	**87.73**	83.05	**60.19**	60.34	**66.03**	**69.75**	**64.95**	**75.60**

**Table 4:** Comparison results of various models.

	Category	PL	BD	BR	GTF	SV	LV	SH	TC	BC	ST	SBF	RA	HA	SP	HC	*mAP*
Model		*AP*															
R^2^CNN (2017)		80.94	65.67	35.34	67.44	59.92	50.91	55.81	90.67	66.92	72.39	55.06	52.23	55.14	53.35	48.22	60.67
RRPN (2018)		88.52	71.20	31.66	59.30	51.85	56.19	57.25	90.81	72.84	67.38	56.69	52.84	53.08	51.94	53.58	61.01
RoI- Transformer (2019)		88.64	78.52	43.44	**75.92**	68.81	73.68	83.59	90.74	77.27	81.46	58.39	53.54	62.83	58.93	47.67	69.56
CADNet (2019)		87.80	82.40	49.40	73.50	71.10	63.50	76.60	**90.90**	79.20	73.30	48.40	60.90	62.00	67.00	62.20	69.90
DRN (2020)		89.71	82.34	47.22	64.10	76.22	74.43	85.84	90.57	86.18	84.89	57.65	61.93	**69.30**	69.63	58.48	73.23
ICN (2018)		81.40	74.30	47.70	70.30	64.90	67.80	70.00	90.80	79.10	78.20	53.60	62.90	67.00	64.20	50.20	68.20
RADet (2020)		79.45	76.99	48.05	65.83	65.46	74.40	68.86	89.70	78.14	74.97	49.92	64.63	66.14	71.58	62.16	69.09
SCRDet (2019)		**89.98**	80.65	52.09	68.36	68.36	60.32	72.41	90.85	**87.94**	**86.86**	65.02	66.68	66.25	68.24	65.21	72.61
MFIAR-Net (2020)		89.62	84.03	52.41	70.30	70.13	67.64	77.81	90.85	85.40	86.22	63.21	64.14	68.31	70.21	62.11	73.49
IRetinaNet (2021)		88.70	82.46	52.81	68.75	78.51	81.45	86.41	90.02	85.37	86.31	**65.10**	65.20	67.80	69.29	64.83	75.53
PolarDet (2021)		89.73	**87.05**	45.30	63.32	78.44	76.65	87.13	90.79	80.58	85.89	60.97	**67.94**	68.20	**74.63**	**68.67**	75.02
S2A-Net (2021)		89.11	82.84	48.37	71.11	78.11	78.39	87.25	90.83	84.90	85.64	60.36	62.60	65.26	69.13	57.94	74.12
RODFormer		89.76	79.64	**56.61**	71.57	**78.60**	**85.29**	**89.93**	90.53	87.73	83.05	60.19	60.34	66.03	69.75	64.95	**75.60**

## Data Availability

Data sharing not applicable.
